# A Plasmid-Transposon Hybrid Mutagenesis System Effective in a Broad Range of Enterobacteria

**DOI:** 10.3389/fmicb.2015.01442

**Published:** 2015-12-22

**Authors:** Rita Monson, Debra S. Smith, Miguel A. Matilla, Kevin Roberts, Elizabeth Richardson, Alison Drew, Neil Williamson, Josh Ramsay, Martin Welch, George P. C. Salmond

**Affiliations:** Department of Biochemistry, University of CambridgeCambridge, UK

**Keywords:** plasposon, transposon mutagenesis, Enterobacteria, *Dickeya*, *Serratia*, plant pathogen, gas vesicles, oocydin A

## Abstract

Random transposon mutagenesis is a powerful technique used to generate libraries of genetic insertions in many different bacterial strains. Here we develop a system facilitating random transposon mutagenesis in a range of different Gram-negative bacterial strains, including *Pectobacterium atrosepticum, Citrobacter rodentium, Serratia* sp. ATCC39006, *Serratia plymuthica, Dickeya dadantii*, and many more. Transposon mutagenesis was optimized in each of these strains and three studies are presented to show the efficacy of this system. Firstly, the important agricultural pathogen *D. dadantii* was mutagenized. Two mutants that showed reduced protease production and one mutant producing the previously cryptic pigment, indigoidine, were identified and characterized. Secondly, the enterobacterium, *Serratia* sp. ATCC39006 was mutagenized and mutants incapable of producing gas vesicles, proteinaceous intracellular organelles, were identified. One of these contained a β-galactosidase transcriptional fusion within the gene *gvpA1*, essential for gas vesicle production. Finally, the system was used to mutate the biosynthetic gene clusters of the antifungal, anti-oomycete and anticancer polyketide, oocydin A, in the plant-associated enterobacterium, *Dickeya solani* MK10. The mutagenesis system was developed to allow easy identification of transposon insertion sites by sequencing, after facile generation of a replicon encompassing the transposon and adjacent DNA, post-excision. Furthermore, the system can also create transcriptional fusions with either β-galactosidase or β-glucuronidase as reporters, and exploits a variety of drug resistance markers so that multiple selectable fusions can be generated in a single strain. This system of various transposons has wide utility and can be combined in many different ways.

## Introduction

Since their initial discovery, transposable elements have greatly assisted our understanding of eukaryotic and prokaryotic genetics. Though transposable elements were first identified and studied in maize, they have been found in virtually all organisms, such as *Drosophila melanogaster, Escherichia coli*, and *Homo sapiens* (McClintock, 1956; Craig, 1990; Frost et al., 2005; Cordaux and Batzer, 2009). Transposons in bacteria can also move from the chromosome to a plasmid or bacteriophage genome, driving genetic evolution and contributing to the spread of antibiotic resistance gene clusters between different bacteria (Jimenez and Davies, 1980; Berg and Berg, 1983).

Advances in molecular genetics and gene cloning have allowed development of genetic tools derived from mobile elements. For example, engineered mobile elements (transposons) have been used in genetic analysis to generate random insertions within the chromosome of a target organism (Berg and Berg, 1983) and insertion of a transposon near, or within, a gene can alter or destroy its function.

Transposons can jump between any genetic elements in their bacterial hosts: chromosome, plasmids or phage genomes (Berg and Berg, 1983). The insertion or excision of a transposon is facilitated by a transposase protein, which is usually encoded within the transposon itself. However, when expression of the transposase protein is decoupled from the transposon, a system can be engineered to generate immobile single transposon insertions in target DNA of interest.

Here we describe the creation of a type of transposon system that has a broad host range within the Enterobacteria that complements existing systems (Dennis and Zylstra, 1998; Larsen et al., 2002). This plasmid-transposon (or plasposon) hybrid system, has been engineered so that it carries an origin of replication, and so, can form a replicating plasmid if excised and self-ligated. Such a plasmid can be sequenced and the transposon insertion site identified with ease. We also used a second method to identify the transposon insertion sites, which relied upon Random Primed PCR (RP-PCR; Jacobs et al., 2003; Fineran et al., 2005). These two parallel methods facilitate quick and straightforward identification of any transposon insertion within a host's genome.

Three test studies were conducted to demonstrate the efficacy of this system. Firstly, the plasposon system described above was used to mutagenise the plant pathogen *Dickeya dadantii*, a cause of soft rot in many different plant species and a pathogen of the aphid *Acyrthosiphon pisum* (Grenier et al., 2006). Secondly, the system was used to create stable transcriptional fusions within *gvpA1*, the first gene in the gas vesicle operon of *Serratia* sp. 39006 (S39006). Thirdly, transposon mutants interrupting the oocydin A biosynthetic gene clusters in *Dickeya solani* MK10 were isolated—one of them generating a transcriptional fusion within the large *oocJ-W* operon. Together, these three studies demonstrate the wide utility of this system for genetic screens in a broad range of bacterial species.

## Materials and methods

### Bacterial strains, plasmids, and growth conditions

A list of the bacterial, fungal and bacteriophage strains and plasmids used in this study can be found in Table 1. All strains were grown in Luria Broth (LB; 5 g l^−1^ yeast extract, 10 g l^−1^ tryptone and 5 g l^−1^ NaCl) in liquid culture (either in 30 mL sealed plastic universal tubes (Thermo Scientific Cat No 128A/P) on a rotary wheel or with shaking at 200 rpm) or on solid LB supplemented with 1.5% agar (LBA) unless otherwise indicated. *E. coli* strains were grown at 37 °C with antibiotic supplements where indicated. All other strains were grown at 30 °C. Where indicated, *D. dadantii* was grown in PMB Medium (0.1% (w/v) yeast extract, 0.1% (NH_4_)_2_SO_4_, 1 mM MgSO_4_, 0.5% glycerol, 0.5% polygalacturonic acid, 7 g l^−1^ K_2_HPO_4_, 2 g l^−1^ KH_2_PO_4_), or iron-limiting MM9 Medium (0.3 g l^−1^ KH_2_PO_4_, 0.5 g l^−1^ NaCl, 1.0 g l^−1^NH_4_Cl, 6.0 g l^−1^ NaOH, and 30.24 g l^−1^ PIPES). Bacterial culture optical density (OD_600 nm_) was measured using a Unicam Heλios Spectrophotometer. Antibiotics and supplements were added at the following concentrations: ampicillin, 100 μg ml^−1^; chloramphenicol, 50 μg ml^−1^; tetracycline, 15 μg ml^−1^; kanamycin, 15 μg ml^−1^ (*E. coli* strain β2163) and 50 μg ml^−1^ (*S. plymuthica* A153, *D. solani* MK10 and S39006); erythromycin, 200 μg ml^−1^, and 2-6-diaminopimelic acid (DAPA), 300 μM.

**Table 1:** **Strains and plasmids used in this study**.

**Bacterial strains**	**Genotype**	**References or source**
*Agrobacterium tumefaciens*	Wild type	GPCS Lab Strain Collection
*Bacillus subtilis* sp 168	Wild type	GPCS Lab Strain Collection
*Chromobacterium violaceum*	Wild type	GPCS Lab Strain Collection
*Citrobacter freundii*	Wild type	GPCS Lab Strain Collection
*Citrobacter rodentium*	Wild type	GPCS Lab Strain Collection
*Dickeya dadantii* LA15	Wild type	GPCS Lab Strain Collection
*Dickeya dadantii* 3937	Wild type	JHI Strain Collection
*D. dadantii* 3937, REM392	3937 transposon mutant *vfmE*::Tn-DS1028 *cat*	This study
*D. dadantii* 3937, REM393	3937 transposon mutant *pecS*::Tn-DS1028 *cat*	This study
*D. dadantii* 3937, REM394	3937 transposon mutant *vfmA*::Tn-DS1028 *cat*	This study
*Dickeya solani* MK10	Wild type	JHI Strain Collection
*D. solani* MK10, MK10oocG	MK10 transposon mutant *oocG*::Tn-KRCPN1; oocydin A negative, Km^r^	This study
*D. solani* MK10, MK10oocN1	MK10 transposon mutant *oocN*::Tn-KRCPN1; oocydin A negative, Km^r^	This study
*D. solani* MK10, MK10oocN	MK10 transposon mutant *oocG*::Tn-KRCPN1; oocydin A negative, Km^r^	Matilla et al., 2014
*Erwinia wasabiae*	Wild type	JHI Strain Collection
*Escherichia coli* CC118λ*pir*	Δ(*ara-leu*) *araD* Δ*lacX*74 *galE galK phoA*20 *thi*-1 *rpsE rpoB argE*(Am) *recA1* λ*pir*	Herrero et al., 1990
*Escherichia coli* DH5α	F^−^ϕ80*lacZ*ΔM15 Δ(*lacZYA*-*argF*) U169 *recA*1 *endA*1 *hsdR*17(${\text{r}}_{\text{K}}^{-}$ ${\text{m}}_{\text{K}}^{+}$) *phoA supE*44 λ^−^*thi*-1 *relA*1 *gyrA*96	Invitrogen
*Escherichia coli* β2163	F^−^ RP4-2-Tc::Mu Δ*dapA*::(*erm-pir*)	Demarre et al., 2005
*Pectobacterium atrosepticum* SCRI1043	Wild type	GPCS Lab Strain Collection
*Pectobacterium brasiliensis*	Wild type	JHI Strain Collection
*Pectobacterium carotovorum* sp ATCC39048	Wild type	GPCS Lab Strain Collection
*Serratia marcescens* sp.12	Wild type	GPCS Lab Strain Collection
*Serratia marcescens* sp. 274	Wild type	GPCS Lab Strain Collection
*Serratia* sp ATCC39006	LacA, lac^−^	GPCS Lab Strain Collection
S39006, REM465	S39006 transposon mutant *gvpA1*::Tn-KRCPN1; Kn	This study
*Serratia* sp. MSU97	Wild type	
*Serratia plymuthica* A153	Wild type, rhizosphere isolate	Hökeberg et al., 1997
*S. plymuthica* MMnO2	A153 transposon mutant *oocS*::Tn-KRCPN1*lacZ*; Km^r^	Matilla et al., 2012
*S. plymuthica* MMnO4	A153 transposon mutant *oocQ*::Tn-KRCPN1; Km^r^	Matilla et al., 2012
*S. plymuthica* MMnO9	A153 transposon mutant *oocN*::Tn-KRCPN1; Km^r^	Matilla et al., 2012
*S. plymuthica* MMnO13	A153 transposon mutant *oocJ*::Tn-KRCPN1; Km^r^	Matilla et al., 2012
*S. plymuthica* MMnO14	A153 transposon mutant *oocC*::Tn-KRCPN1; Km^r^	Matilla et al., 2012
*S. plymuthica* MMnO15	A153 transposon mutant *oocU*::Tn-KRCPN1; Km^r^	Matilla et al., 2012
**FUNGI/OOMYCETE STRAINS**		
*Pythium ultimum*	Wild type, plant pathogen	C. A. Gilligan
*Verticillium dahliae* 5368	Wild type, plant pathogen	R. Cooper
**PHAGES**		
φXF3	Generalized transducing phage for *Dickeya solani*	Matilla et al., 2014
φMAM1	Generalized transducing phage for *S. plymuthica* A153	Matilla and Salmond, 2014
φOT8	Generalized transducing phage for S39006	Evans et al., 2010
**PLASMIDS**		
pACYC184	*cat, tetA*	NEB
pBluescriptIIKS+	*bla, lacZ*	Agilent
pDS1028	*tetA, tnp, oriR6K, cat*	This study
pKRCPN1	*tetA, tnp, ‘lacZ, oriR6K, aph*	This study
pBM1001	*cat, lacZ*	This study
pBM1002	*tetA, lacZ*	This study
pACYC177	*aph, bla*	New England Biolabs
pHP45Ω	*aph, bla*	Prentki and Krisch, 1984
pRL27	*aph, oriT, oriR6K, tnp, tetA_*p*_*	Larsen et al., 2002

### DNA manipulations, oligonucleotides, and sequencing

Unless otherwise stated, standard molecular biological methods were used for all DNA manipulations. Plasmid DNA was extracted using a Qiagen Miniprep Kit (Qiagen) or an Anachem Keyprep Kit (Anachem) according to the manufacturer's instructions. Where required, DNA was extracted from individual strains using a Qiagen DNeasy Kit according to manufacturer's instructions. All restriction enzymes used were obtained from New England Biolabs and used according to manufacturer's protocols. DNA fragments were ligated using T4 DNA ligase (NEB). Oligonucleotides were obtained from Sigma Aldrich and are listed in Table 2. DNA sequencing was conducted in the Department of Biochemistry Sequencing Facility, University of Cambridge, Cambridge, United Kingdom.

**Table 2:** **Oligonucleotides used in this study**.

**Name**	**Sequence (5′–3′)**	**Notes**
oMAMV1	GGAATTGATCCGGTGGATG	Sequencing primer pKRCPN1
oMAMV2	GCATAAAGCTTGCTCAATCAATCAC	Sequencing primer pKRCPN1
oREM7	CTAGAGTCGACCTGCAGGC	Sequencing primer pDS1028
oREM8	CACAGGAACACTTAACGGC	Sequencing primer pDS1028
oPF106	GACCACACGTCGACTAGTGCNNNNNNNNNNAGAG	Random prime PCR primer 1
oPF107	GACCACACGTCGACTAGTGCNNNNNNNNNNACGCC	Random prime PCR primer 2
oPF108	GACCACACGTCGACTAGTGCNNNNNNNNNNGATAC	Random prime PCR primer 3
oPF109	GACCACACGTCGACTAGTGC	Random prime adaptor primer
5′ΩPAC	CCCTTAATTAACCGCGAGCTTGGCAC	Amplification of Ω fragment forward
3′ΩERV	CCCGATATCGCGCGAGGCAGAAGC	Amplification of Ω fragment reverse

### Cloning of pDS1028 and pKRCPN1

The plasmid pBM1001 was created by insertion of the XmnI/BstBI fragment from pACYC184 (containing the *aph* gene) into pBluescript II KS+. The plasmid pBM1002 was created by insertion of the StyI/XbaI fragment from pACYC184 (containing the *tetA* gene) into pBluescript II KS+. The plasmid pDS1028 was created in three steps. Firstly, the XbaI/EcoRI fragment containing the tetracycline resistance gene (*tetA*) from pBM1002 was ligated into pRL27 to create plasmid pDS1017. Secondly, pDS1017 was digested with SacI and pBM1001 was digested with AccI to remove the *cat* gene. Both fragments of DNA were treated to create blunt ends and were ligated together to create pDS1022. Finally, oligonucleotides 5′ΩPAC and 3′ΩERV were used to amplify the Ω transcriptional and translational terminator from pHP45Ω, then this fragment was digested with EcoRV/PacI, and ligated into pDS1022 that had been compatibly digested, to create plasmid pDS1028.

The plasmid pKRCPN1 was created by modifying pDS1028. The promoterless *lacZ1* fragment from miniTn5*lacZ1* was ligated into the KpnI site of pDS1028, to create pNRW112. Oligonucleotide pair KR19/KR21 were used to amplify the *aph* gene from pACYC177. This was digested with BstBI and PacI and ligated into compatible sites of digested pNRW112 to create pKRCPN1.

### Transposon mutagenesis

Overnight cultures of each recipient strain and the *E. coli* donor strain were grown at 30 °C and 37 °C, respectively. The donor and recipient strains were mixed together in the ratio indicated. The most efficient ratio for transposon mutagenesis for most strains was 1:3 (donor: recipient). Thirty microlitres of this mixture was spotted onto LBA + DAPA where required, and allowed to dry. The mixture was incubated overnight at 30 °C and the mixed culture then resuspended in 1 ml of sterile LB. The conjugation mixture was serially diluted and plated onto minimal agar [0.2% glucose, 0.41 mM MgSO_4_, 0.1% (NH_4_)_2_SO_4_, 0.7% K_2_HPO_4_, 0.2% KH_2_PO_4,_1.5% agar], caseinase agar (nutrient broth agar with 1% Marvel skim milk, 1.5% agar), or LBA, with appropriate antibiotic selection, and incubated for 48 h at 25 °C (*D. dadantii* and *D. solani* MK10) or 30 °C (S39006, *S*. *plymuthica* A153). Putative mutants were then tested for acquisition of the full plasmid by patching a selection of colonies onto LBA + Tc.

### Identification of transposon insertion sites by replicon cloning

DNA was extracted from transposon mutants and digested with an enzyme that does not cut within the transposon (See Table 3 for a full list). Digested DNA was purified using an Anachem Spin PCR Clean Up Kit then self-ligated. The ligation mixture was then used to transform *E. coli* strains CC118λ*pir* or β2163 by heat shock and plated onto LBA (containing DAPA for β2163) and the appropriate antibiotic. Replicon DNA was subsequently isolated by plasmid extraction and the precise transposon insertion site identified by sequencing using either oREM7 or oREM8 primers.

**Table 3:** **Enzymes that do not cut pDS1028 or pKRCPN1**.

**Enzymes**	**Cut site**
**Enzymes that do not cut either pDS1028 or pKRCPN1**	
AflII	Cgˇ TTA_ˆ_ G
ApaI	G_ˆ_ GGCCˇ C
AvrII	Cˇ CTAG_ˆ_ G
BbvCI	CCˇ TCA_ˆ_ GC
BstEII	Gˇ GTNAC_ˆ_ C
FseI	GG_ˆ_ CCGGˇ CC
PflFI	GACNˇ N_ˆ_ NGTC
PmeI	GTTTˇ _ˆ_ AAAC
PmlI	CACˇ _ˆ_ GTG
PspOMI	Gˇ GGCC_ˆ_ C
StuI	AGGˇ _ˆ_ CCT
SwaI	ATTTˇ _ˆ_ AAAT
Tth111I	GACNˇ N_ˆ_ NGTC
XmnI	GAANNˇ _ˆ_ NNTTCC
**Enzymes that do not cut pKRCPN1 but cut pDS1028**	
NcoI	Cˇ CATG_ˆ_ G
ScaI	AGTˇ _ˆ_ ACT
SpeI	Aˇ CTAG_ˆ_ T
**Enzymes that do not cut pDS1028 but cut pKRCPN1**	
AflII	Cˇ TTAA_ˆ_ G
ApaLI	Gˇ TGCA_ˆ_ C
BclI	Tˇ GATC_ˆ_ A
BsrGI	Tˇ GTAC_ˆ_ A
BssSI	Cˇ ACGA_ˆ_ G
MluI	Aˇ CGCG_ˆ_ T
NsiI	A_ˆ_ TGCAˇ T
PciI	Aˇ CATG_ˆ_ T
PvuI	CGˇ AT_ˆ_ CG
ZraI	GACˇ _ˆ_ GTC

### Identification of transposon insertion sites by RP-PCR

RP-PCR was conducted largely as previously described (Jacobs et al., 2003; Fineran et al., 2005). Briefly, DNA from transposon mutants was amplified using a two-step PCR process. In the first round, DNA was amplified using a random oligonucleotide mix (oPF106, oPF107, oPF108) and the transposon specific oligonucleotide (See Table 2 for examples). DNA from this reaction was used in a second amplification using oPF109 and a second transposon specific oligonucleotide. The resulting DNA fragments were amplified with the appropriate transposon specific oligonucleotide.

### Phenotypic plate assays

*D. dadantii* mutants were screened on agar plates for production of protease, cellulase, swimming motility, pectate lyase and siderophores as described previously (Cubitt et al., 2013; Monson et al., 2013). Briefly, a normalized number of *D. dadantii* cells was spotted in 10 μl onto siderophore agar (Schwyn and Neilands, 1987), pectate lyase agar (Pemberton et al., 2005), cellulase agar (Pemberton et al., 2005), gelatinase agar (Burr et al., 2006), caseinase agar (Cubitt et al., 2013), or swimming motility agar (Monson et al., 2013). Swimming plates were incubated for 14 h and all other plates were incubated at 25 °C for 2 days. Swimming plates, caseinase plates and siderophore plates required no further development and were analyzed visually. Cellulase, gelatinase and pectate lyase plates were developed appropriately (Cubitt et al., 2013; Monson et al., 2013). Antagonistic activities of bacterial strains against the oomycete, *Pythium ultimum* and the fungus, *Verticillium dahliae*, were assayed as described previously (Matilla et al., 2012).

### Indigoidine liquid assay

Production of indigoidine was assessed in liquid as described previously (Reverchon et al., 2002). Briefly, cells were grown in PMB, LB, or MM9, pelleted by centrifugation, supernatant samples removed and the cell pellets snap frozen in liquid nitrogen. Cells were thawed on ice, the pellet resuspended in 1 ml dimethyl sulfoxide (Sigma) and vortexed. Cellular debris was pelleted by centrifugation (10 000 *g*, 10 min) and the A_615_ of the supernatant measured. Indigoidine levels were expressed as the A_615_ OD_600_^−1^. Where appropriate, a student's *t*-test was used to determine statistical significance of differences between mutants.

### Flotation assay

Flotation assays of S39006 were carried out as described previously (Ramsay et al., 2011). Briefly, strains were grown in a 30 ml sealed universal plastic tube overnight at 30 °C in LB. The following day, a normalized number of cells were used to inoculate a 5 ml culture which was grown for 24 h on a roller wheel at 30 °C. Cultures were left to settle at room temperature for 24 h.

### Microscopy

Samples were taken directly from liquid culture without further preparation, largely as described previously (Ramsay et al., 2011). Phase contrast light microscopy was undertaken using an Olympus BX-51 microscope with a 100x oil immersion lens. Images were captured using a QICAM camera and QCapture Pro software. Images were cropped and the scale bar added using Adobe Photoshop. All images were representative of those observed for any particular strain.

### β-galactosidase assay

β-galactosidase activity was determined by monitoring the breakdown of 4′-Methylumbelliferyl-β-D-galactopyranoside (Melford Laboratories). At the indicated time point, samples of liquid culture (100 μl) were taken and frozen at −80°C until needed. Samples were thawed and 10 μl removed and frozen at −80°C for 15 min and thawed at 37 °C. Next, 100 μl of Reaction Mix (Phosphate-buffered saline, 400 μg ml^−1^ lysozyme, 250 μg ml^−1^ 4′-Methylumbelliferyl-β-D-galactopyranoside) was added to the samples and they were monitored in a Gemini XPS plate reader using the following parameters: 360 nm excitation, 450 nm emission, 435 nm cut off, eight reads per well, measured every minute for 30 min. Relative fluorescence units (RFU) per min were calculated during a linear phase of fluorescence increase and were normalized to the OD_600_ creating an activity measurement of RFU OD_600_^−1^.

### Sequence information

The sequences of pKRCPN1 and pDS1028 were deposited in Genbank and given the accession numbers KT991288 and KT991389, respectively.

## Results

### The plasmids pDS1028 and pKRCPN1 are capable of mutagenizing some gram-negative bacteria

**Figure 1:**
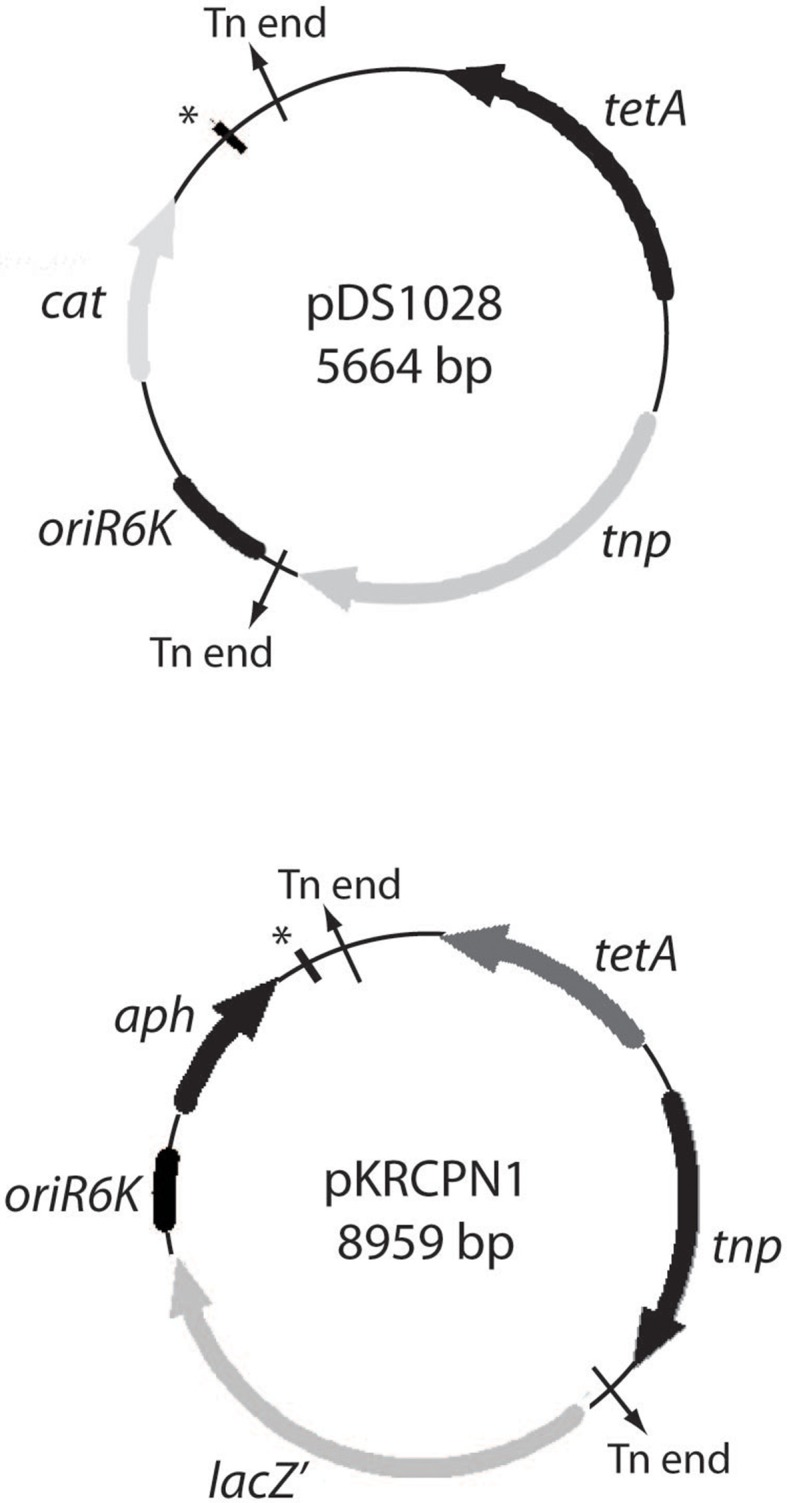
**Plasmid maps of pDS1028 and pKRCPN1**. Plasmid maps of pDS1028 (top) and pKRCPN1 (bottom) were created. The open reading frames present in each plasmid are indicated as thick arrows with the name adjacent. The transposon ends are indicated with black arrows pointing out of the plasmid. The Ω fragment is indicated with a thick black line and a ^*^. The length of each plasmid in basepairs (bp) is indicated inside the plasmid.

The pDS1028 and pKRCPN1 plasmids were created as described in Materials and Methods. Both plasmids were sequenced and a schematic of them is shown in Figure 1. Though both pDS1028 and pKRCPN1 contain the transposase gene to facilitate transposition, it is not located within the transposon itself. Thus, when the transposon has “hopped” into a chromosomal location, if the plasmid is unable to replicate in this host (all *pir*^−^ strains) a stable insertion will be created. These two systems were then used to isolate gene knockouts or transcriptional gene fusions. Furthermore, as the two transposons contain different antibiotic selections, they can also be used in combination. In addition, a version of this transposon, described by Ramsay and colleagues has been engineered with a *uidA* cassette in place of the *lacZ* cassette described in this work (Ramsay et al., 2011).

The utility of pDS1028 and pKRCPN1 was assessed in 20 different bacterial host strains. Different ratios of *E. coli* donor and recipient cells were tested and putative transposon insertions identified. A tetracycline resistance cassette was encoded in the plasmid backbone of both pDS1028 and pKRCPN1 but not within the transposon. Therefore, in the case of pDS1028, if the full plasmid transferred between the donor and recipient strain, any resulting mutants would be resistant to both chloramphenicol and tetracyline, and any transposon insertions would be resistant to chloramphenicol but sensitive to tetracycline. Of the 20 strains tested using both plasposon systems, putative transposon insertions were observed in 16 strains (Table 4). For each strain where putative transposon insertions were observed, 50 colonies were tested for tetracycline sensitivity. No tetracycline resistant colonies were observed for any strain with successful transposon insertions; therefore we concluded that our mutants were transposon insertions and not the result of plasmid transfer.

**Table 4:** **Transposon mutant efficiency**.

**Test strain**	**Optimal ratio (Donor: Recipient)**	**Transposon mutant frequency**
*Agrobacterium tumefaciens*	3: 1	<2.63 × 10^−9^
	2: 1	<3.45 × 10^−9^
	1: 1	<2.43 × 10^−9^
	1: 2	<6.34 × 10^−9^
	1: 3	<3.88 × 10^−9^
*Bacillus subtilis* sp 168	3: 1	<7.34 × 10^−8^
	2: 1	<1.85 × 10^−9^
	1: 1	<1.75 × 10^−9^
	1: 2	<9.18 × 10^−8^
	1: 3	<2.87 × 10^−9^
*Chromobacterium violaceum*	1: 3	3.44 × 10^−5^
*Citrobacter freundii*	1: 1	2.38 × 10^−5^
*Citrobacter rodentium*	1: 1	1.84 × 10^−5^
*Dickeya dadantii* LA15	1: 1	2.33 × 10^−5^
*Dickeya dadantii* 3937	1: 3	5.21 × 10^−5^
*Dickeya solani*	1: 3	3.81 × 10^−6^
*Erwinia wasabiae*	1: 3	2.59 × 10^−5^
*Pectobacterium atrosepticum* SCRI1039	1: 3	8.45 × 10^−6^
*Pectobacterium atrosepticum* SCRI1043	1: 3	9.17 × 10^−6^
*Pectobacterium brasiliensis*	1: 3	1.83 × 10^−7^
*Pectobacterium carotovorum* Attn10	1: 3	3.24 × 10^−5^
*Pectobacterium carotovorum* sp 193	1: 3	7.87 × 10^−7^
*Pectobacterium carotovorum* sp ATCC39048	1: 1	1.19 × 10^−6^
*Serratia marcescens* 12	1: 2	6.67 × 10^−5^
*Serratia marcescens* 274	1: 2	3.79 × 10^−6^
*Serratia plymuthica A153*	1:3	2.36 × 10^−5^
*Serratia* sp 39006	1: 3	2.41 × 10^−6^
*Serratia* sp. MSU97	3: 1	<3.85 × 10^−9^
	2: 1	<6.28 × 10^−9^
	1: 1	<2.56 × 10^−9^
	1: 2	<3.26 × 10^−9^
	1: 3	<4.95 × 10^−9^

We were unable to detect putative transposon insertions in *Serratia marcescens* MSU97 or the Gram-positive, *Bacillus subtilis*. We tested for the presence of *E. coli* donor cells after an 8 h incubation with *S. marcescens* MSU97 and no donor cells were detected (data not shown). MSU97 produces potent bioactive compounds, potentially killing donor cells during conjugal mating (Matilla et al., 2012). Therefore, the lack of transposon mutants from MSU97 may have been due to killing of the *E. coli* donor strain, making successful conjugation unlikely. We were also unable to detect any transposon insertions in *B. subtilis*, though unlike *S. marcescens* MSU97, this was not due to donor cell death (data not shown). *B. subtilis* is a Gram-positive organism and conjugation with the Gram-negative *E. coli* donor strain was not successful.

**Figure 2:**
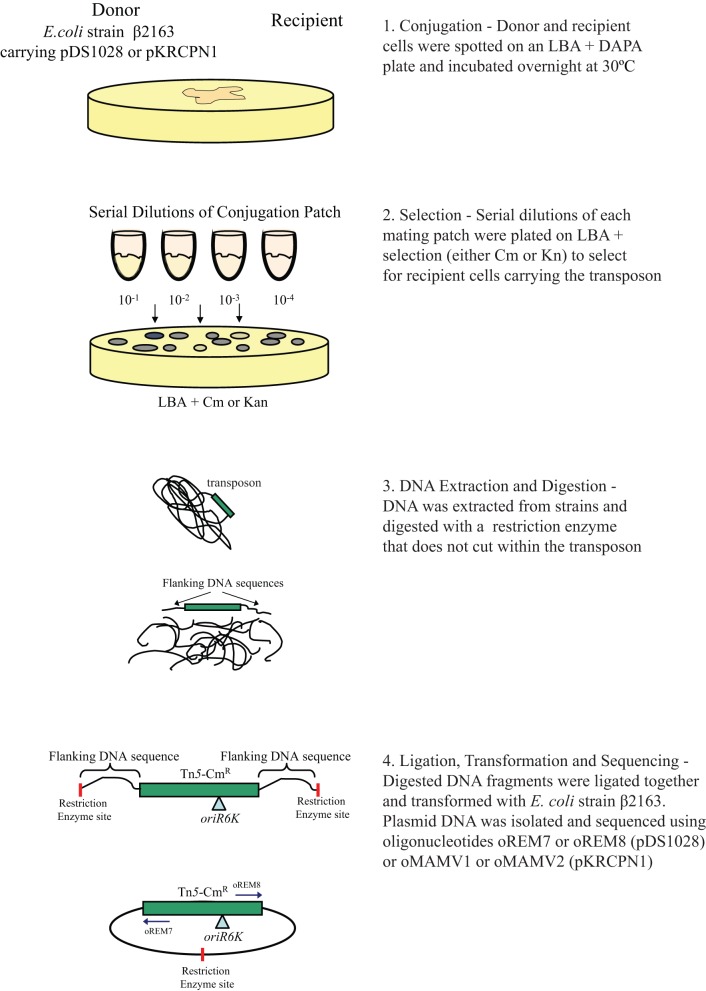
**Schematic showing the procedure for isolating transposon mutants**.

In the remaining 17 strains, putative transposon mutants were detected with high frequency in all strains except *Agrobacterium tumefaciens* using both pKRCPN1 and pDS1028 (Table 4). For almost all strains, the optimal ratio of donor to recipient cells was 1:3 (Table 2). To assess randomness and whether the plasposon system could be used to isolate mutants with particular traits, we performed three pilot studies (i) in the plant pathogen *D. dadantii* strain 3937; (ii) in the enterobacterium S39006; and (iii) in two oocydin A producing strains, the biocontrol rhizobacterium, *Serratia plymuthica* A153, and the phytopathogen, *D. solani* MK10. A schematic showing the full model of how transposon mutagenesis was performed, and mutants were identified is shown in Figure 2.

### Mutations in the *vfm* operon in *D. dadantii* are defective for protease production

*D. dadantii* 3937 was conjugated with *E. coli* CC118λ*pir* carrying pDS1028. Following conjugation, transposon insertions were screened on caseinase agar containing chloramphenicol (to select for the presence of the transposon), and casein degradation was assessed by scoring for clearing around colonies. Over 15,000 random transposon insertion mutants were screened for reduced caseinase activity, and 10 mutants were identified. Of these 10 mutants, we chose to continue characterization of two mutants: REM392 and REM394.

The transposon encoded within pDS1028 also contains an origin of replication: *oriR*6K. As a result, genomic DNA from any transposon mutant can be digested, with a restriction endonuclease that does not cut within the transposon and can then be self-ligated to form a plasmid that will form a replicon in a *pir*^+^ strain. Genomic DNA from REM392 was digested with NsiI, self-ligated, and used to transform *E. coli* strain CC118λ*pir*. Using oligonucleotides internal to the transposon, the sequence adjacent to the transposon was identified. By applying this method, we identified an insertion in *vfmE* (REM392) and *vfmA* (REM394) that caused reduced caseinase (or protease) production in these strains.

**Figure 3:**
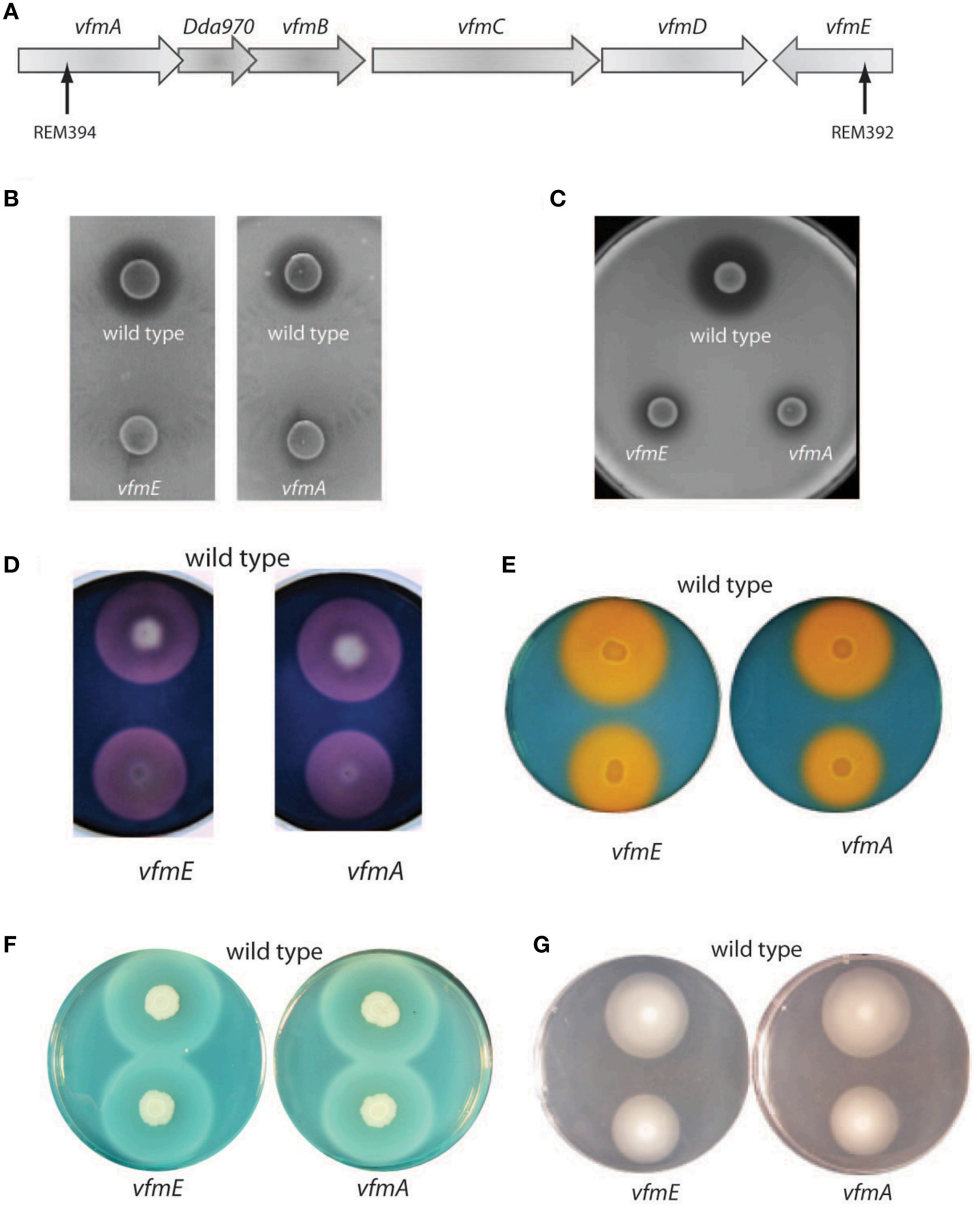
**Mutations in the *vfm* region in *D. dadantii***. **(A)** Schematic representation of the *vfm* cultures in *D. dadantii*. Genes are represented as open arrows. Black arrows within *vfmA* and *vfmE* indicate the sites of the transposon insertion in REM394 and REM392, respectively. Caseinase **(B)** and gelatinase **(C)**, cellulase **(D)**, siderophore **(E)**, pectate lyase **(F)**, and swimming **(G)** assay plates comparing wild type and *vfmE* or *vfmA* mutants. For each strain, 5 μl of normalized overnight culture was spotted onto the plate and grown at 25 °C for 18 h (swimming) or 48 h (all other plates). On each plate, the halo surrounding the cell is representative of the enzymatic activity (cellulase and pectate lyase), the siderophores produced or flagellum based swimming.

VfmE is a transcriptional regulator of the AraC family and was previously identified as an activator of virulence determinants in *D. dadantii* (Nasser et al., 2013). We also identified a mutation in *vfmA*, the first gene in one operon of the *vfm* region, and disrupts the function of five genes: *vfmA, vfmZ, vfmB, vfmC*, and *vfmD* through polarity (Figure 3). VfmA shares high levels of homology with 3-oxoacyl-acyl-carrier-proteins, a subclass of decarboxylating condensing enzymes that include polyketide synthases, though the full role of this protein has not been studied in *D. dadantii* (Nasser et al., 2013).

To characterize these mutations further, each mutant was tested for protease, cellulase and pectate lyase production (Figure 3). We also examined siderophore production and swimming motility in each of mutant strains. Swimming motility was reduced in both of the mutants tested (Figure 3G). Protease and cellulase production were also reduced in both mutants. Siderophore production was slightly reduced in both of the mutants tested and we were unable to detect a difference in pectate lyase production between mutant and wild type *D. dadantii* using plate assays (Figure 3).

### A mutation in *pecS* results in production of the pigment indigoidine

During our screen of *D. dadantii* mutants, we also identified a mutant that appeared darker on caseinase plates. *D. dadantii* is known to have the cryptic capacity to produce the blue pigment indigoidine, though not naturally under standard laboratory conditions (Reverchon et al., 2002). On LB agar rather than caseinase agar, REM393 still produced the blue pigment (data not shown) and we concluded that this blue pigment was likely indigoidine and the transposon insertion in this strain led to cryptic activation.

**Figure 4:**
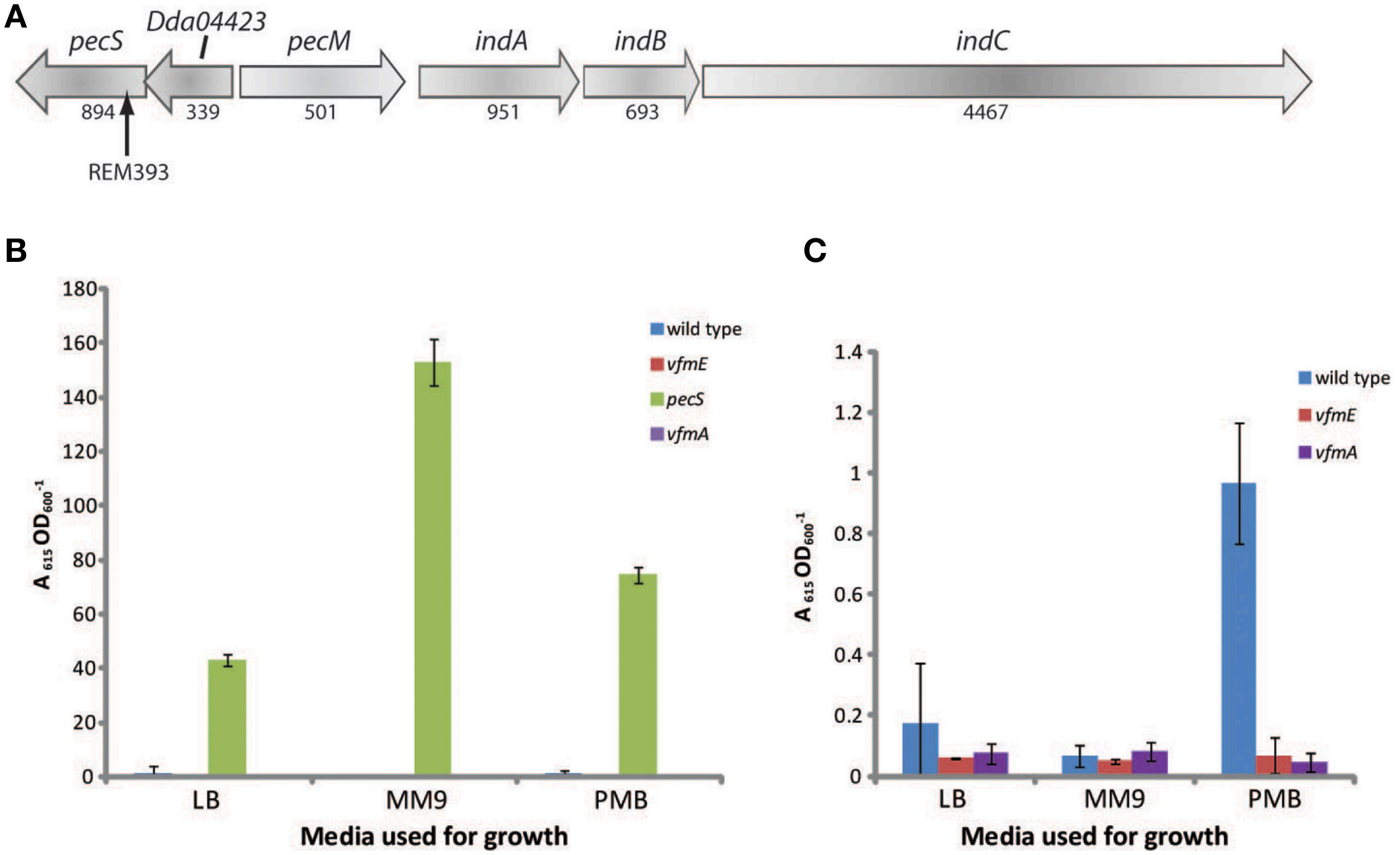
**A mutation in *pecS* overproduces indigoidine**. **(A)** Schematic representation of the *pecS* and indigoidine biosynthesis genetic cluster in *D. dadantii*. Genes are represented as open arrows and their length in basepairs is indicated below each gene. An arrow indicates the site of the transposon insertion in REM393. **(B,C)** Indigoidine production in different media. Wild type, *vfmE, vfmA*, and *pecS* strains were grown in the indicated liquid media and after 16 h indigoidine production assayed. Values represent the average of three independent replicates. The data presented in **(C)** are the same as in **(B)** but with *pecS* removed, the scaling reflects this change. Error bars indicate ±SD (*n* = 3).

Previous work demonstrated that the transcriptional regulator PecS represses production of indigoidine under standard laboratory growth conditions (Reverchon et al., 2002). Therefore, we examined whether the transposon insertions in this strain was located within *pecS*. Using RP-PCR, we determined that the transposon insertion within REM393 was located within *pecS* (Figure 4). Mutations in *pecS* have been characterized previously (Reverchon et al., 2002), but we wanted to show quantitatively that indigoidine production was activated (or derepressed) in REM393, compared with wild type. Pigment levels were assayed in wild type and REM393 in different growth media. Indigoidine levels in wild type cells were significantly lower than in a *pecS* mutant. We also tested indigoidine levels in the *vfmA* and *vfmE* mutants. In both of these strains, indigoidine levels were significantly less than wild type when grown in PMB media, suggesting a link between the *vfm* region and indigoidine production.

### Identification of a transcriptional fusion in *gvpA1* in S39006

**Figure 5:**
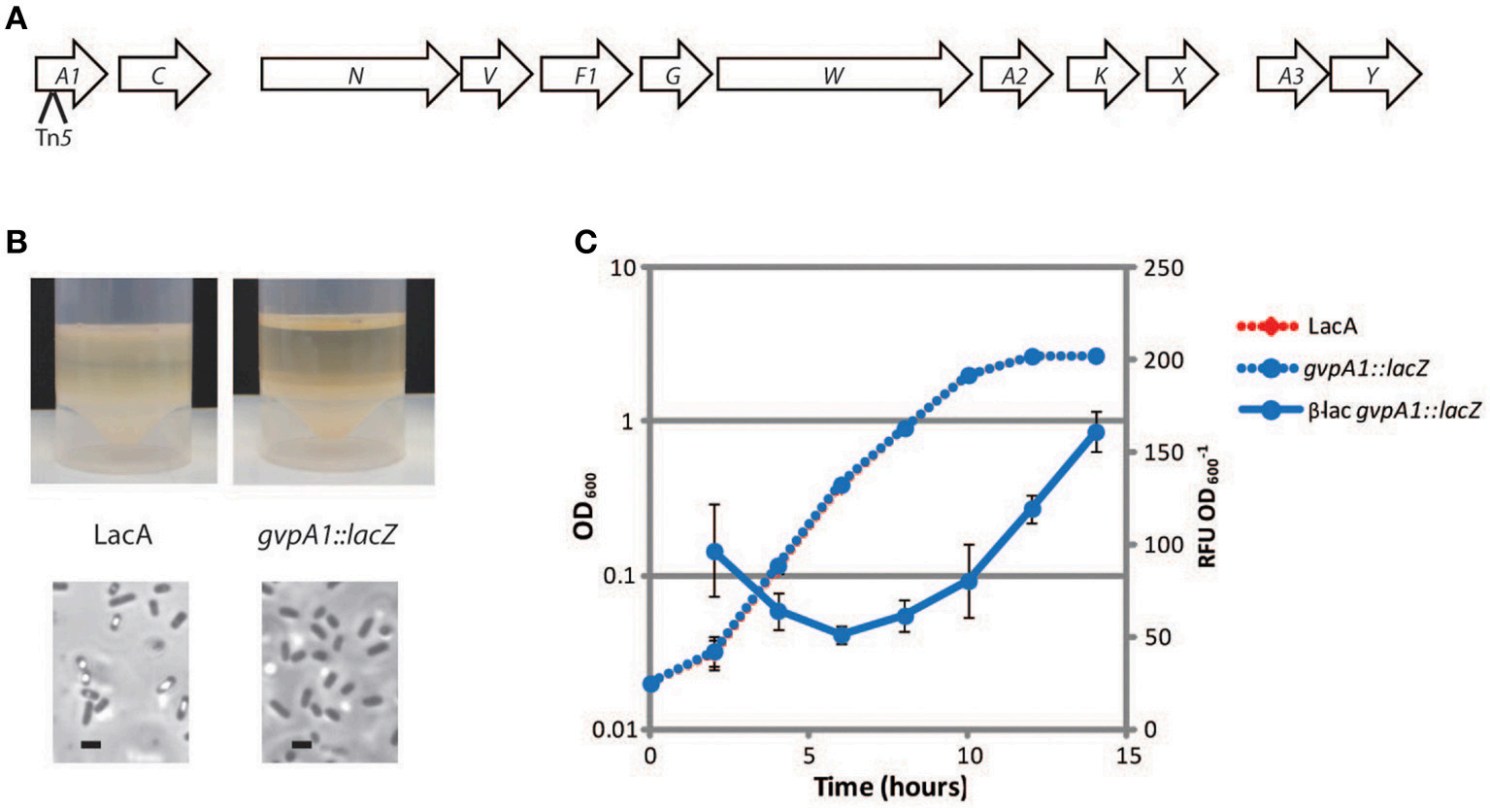
**Isolation of a mutant containing a transcriptional fusion in *gvpA1* that fails to produce GVs**. **(A)** The genetic organization of the GV operon starting with *gvpA1*. Each gene within the operon is indicated with an arrow. All gene names have been shortened and “gvp” has been removed. The site of the transposon insertion is indicated with an arrow below *gvpA1*. **(B)** Floatation assays of wild type *Serratia* 39006 and *gvpA1* (top) and PCM images of the same cells (bottom). Scale bars indicate 1 μm. **(C)** Activity of *gvpA1::lacZ* fusion throughout growth. Wild type (red dashed) and *gvpA1::lacZ* (blue dashed) cultures were monitored throughout growth. β-galactosidase (β-lac) activity was determined from samples taken at each time point (blue solid line). Values indicate the average of three independent replicates. Error bars indicate ±SD.

S39006 produces gas vesicles, proteinaceous intracellular organelles that are permeable to gas but not liquids. These organelles facilitate buoyancy in a static water column, allowing cells to colonize the air-liquid interface (Ramsay et al., 2011). Gas vesicles are visible in cells by phase contrast microscopy, as they conglomerate as a light-refractile gas “vacuole” inside the cell. S39006 colonies appear opaque on plates due to the production of gas vesicles and colonies incapable of producing gas vesicles appear translucent. To identify mutants that lack gas vesicles, S39006 was mutagenized using the donor plasposon pKRCPN1 and translucent (presumptive gas vesicle defective) strains were identified. We were particularly interested in identifying transcriptional fusions of the β-galactosidase gene to *gvpA1*, the first gene in the primary gas vesicle synthesis operon. Over 5000 colonies were screened and a single translucent colony, containing the transposon 88 bp 3′ of the *gvpA1* translational start site, in the correct orientation to form a transcriptional fusion, was identified (Figure 5A). To confirm that the phenotypes were the result of this transposon alone, mutations were moved into a clean genetic background using the generalized transducing phage φOT8 followed by phenotypic confirmation in the transductants. β-galactosidase activity (a proxy for *gvpA1* transcription) was monitored throughout growth and showed similar activity to β-glucuronidase fusions in the same gene that were reported by Ramsay et al. (2011; Figure 5). Thus, the plasposon system produces stable insertions and gene fusions in S39006.

### Mutagenesis of the oocydin A gene clusters in *serratia* and *dickeya* strains

**Figure 6:**
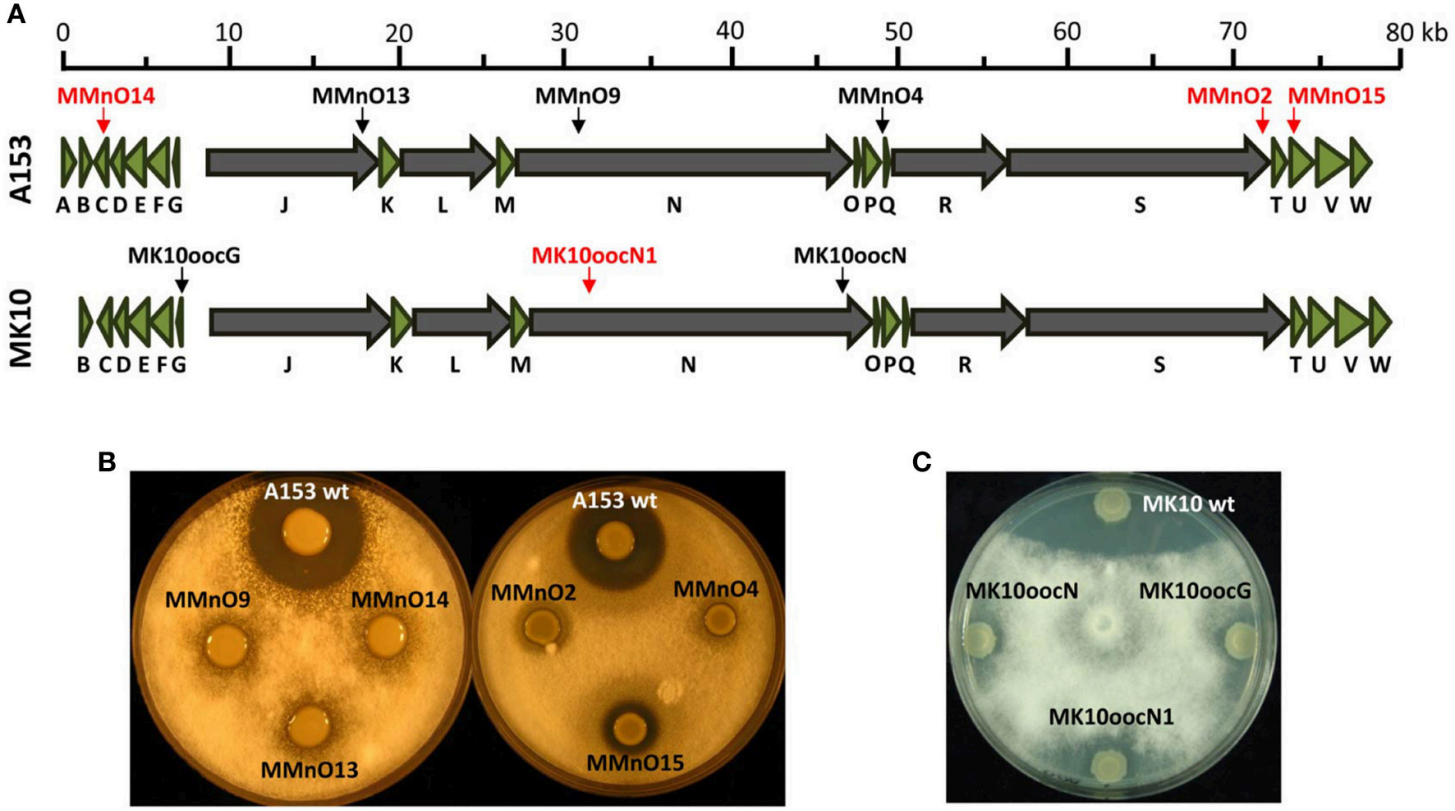
**Isolation of oocydin A-defective mutants using the plasposon pKRCPN1. (A)** Genetic organization of the *ooc* biosynthetic gene cluster in *Serratia plymuthica* A153 and *Dickeya solani* MK10. Multidomain polyketide synthase genes are shown in gray. Arrows indicate the location of the Tn-KRCPN1 transposon insertions and red arrows indicate that the transposon has been inserted in the right orientation, therefore generating a transcription fusion. The *oocA* gene is absent from the *ooc* gene clusters in all *Dickeya* strains. **(B)** Antifungal activity of *S. plymuthica* A153 strains against *Verticillium dahliae*. MmnO15 produce none and 3–5% of the oocydin A wild type levels, respectively (Matilla et al., 2012). **(C)** Anti-oomycete bioactivities of *D. solani* MK10 strains toward *P. ultimum*. The bioassays were repeated at least three times, and representative results are shown. *P. ultimum* and *V. dahliae* pictures were taken after 48 and 96 h of incubation at 25 °C, respectively.

The halogenated haterumalide, oocydin A, was initially isolated from a plant epiphytic bacterial strain due to its strong bioactivity against plant-pathogenic oomycetes (Srobel et al., 1999). However, oocydin A has been also shown to possess antifungal (Thaning et al., 2001), anticancer (Takada et al., 1999), and anti-hyperlipidemic (Sato et al., 2005) properties. Previously, a random transposon mutant library in the oocydin A producing strain, *S. plymuthica* A153 was constructed and mutants defective in oocydin A production were isolated. We found recently that the *ooc* gene cluster is widely distributed within the *Dickeya* genus, including the aggressive phytopathogen *D. solani* MK10 (Matilla et al., 2015). Here, we employed the plasposon pKRCPN1 to isolate two oocydin A-defective mutants in the phytopathogen *D. solani* MK10, MK10oocG, and MK10oocN1 (Figures 6A,C). The transconjugant library of MK10 was screened for mutants defective in bioactivities against the plant-pathogenic fungi and oomycete, *V. dahliae* and *P. ultimum*, respectively (Figures 6B,C).

The transducing bacteriophage ϕXF3 was used to confirm that the observed phenotypes (Figure 6C) were associated with single transposon insertions (Matilla et al., 2014). One of the of the isolated mutants, strain MK10oocG, formed a transcriptional fusion which will allow the study of the expression of the large *oocJ-W* operon (Figure 6A).

## Discussion

Random transposon mutagenesis has wide ranging usages in many different bacterial systems. This work demonstrates the utility of the plasposon system in different hosts, in particular, by creating transcriptional fusions which can be combined together easily. Firstly, we isolated mutants in *D. dadantii* defective in protease production and with cryptic activation of indigoidine production. We also used this system to create a stable transcriptional fusion in *gvpA1*, the first gene in the gas vesicle cluster of S39006, allowing transcriptional quantification of the gas vesicle operon throughout growth. Finally, we identified mutants in the *ooc* gene cluster in the phytopathogenic bacterium *D. solani* MK10.

The plasposon system described in this work is not the first of its kind (Dennis and Zylstra, 1998), but allows any transposon insertion to be identified in two ways, either through what we term “Random Primed PCR” (RP-PCR) or through the creation of a replicon using the origin of replication found within the transposon itself. The use of two different plasposon selection systems allows increased flexibility, e.g., the construction of double mutants, making this a useful resource when engineering targeted mutants. Often one of the most laborious stages of a transposon mutagenesis is the identification of individual insertion sites. The plasposon system described here allows for identification by two different methods, an important feature should any single technique fail to provide a clean answer.

Our pilot studies also yielded interesting results that demonstrated the efficacy of our system. The *vfm* region of *D. dadantii* has been identified as important for virulence (Nasser et al., 2013). This operon is responsible for production of a new, as yet unidentified, intercellular signal. Here we identified transposon insertion in two of the four transcriptional units comprising the *vfm* region, *vfmE*, and *vfmA-vfmD*. Tests by Nasser and colleagues showed that mutants defective in both of these transcriptional units showed reduced virulence in the *Saintpaulia ionantha* (African violet) plant model (2013). We found similar results in our analysis of pectate lyase and protease production in our transposon mutants (though not for cellulase production, where our *vfmE* and *vfmA* mutants did not produce significantly less cellulase activity). This may be due to differences in the assays that were used.

The cryptic pigment indigoidine is not expressed under normal laboratory conditions in *D. dadantii* (Reverchon et al., 2002). We identified a mutant (REM393), within *pecS*, that produced indigoidine. The *pecS* mutant showed increased pectate lyase activity, cellulase activity and siderophore production. In media containing glycerol as a carbon source (MM9), we saw a significant increase in indigoidine (Figure 4). These observations are consistent with the results of Reverchon et al. (2002) who demonstrated that *pecS* acts as a repressor of indigoidine.

We also examined indigoidine production in our *vfmA* and *vfmE* mutants. Though indigoidine is not normally produced in standard laboratory media, we observed small amounts of production in media containing polygalacturonic acid (PMB), a degradation product of plant cell walls—and perhaps a mimic of *in planta* conditions. However, in a *vfmA* or *vfmE* mutant, no such production was observed, suggesting that the metabolite or putative signaling molecule produced from this region may somehow induce indigoidine production in addition to affecting plant virulence (Nasser et al., 2013).

The identification of biosynthetic clusters for different antifungal compounds is of great interest. From previous work, the genetic cluster responsible for production of the halogenated haterumalide, oocydin A, was identified in A153 (Matilla et al., 2012). From bioinformatic analysis, we knew that this cluster was also present in the phytopathogen *D. solani* MK10. Using the plasposon system, two transposon insertion mutants within the MK10 cluster were identified, one a transcriptional fusion, that could be used to examine transcriptional changes of the cluster under different environmental conditions.

The primary purpose of this study was to construct and determine the general utility of the plasposon system. We believe that these constructs will have wide utility in studies of Gram-negative bacteria. High rates of conjugation were observed in most Gram-negative strains (unless donor killing was a problem). This system will improve functional genomics in *Serratia, Pectobacterium, Citrobacter, Dickeya*, and *Chromobacterium species*, but this was a limited repertoire of test hosts. It is very likely that this plasposon system will have far wider utility in taxonomically distant Gram-negative bacteria.

## Author contributions

RM, DS, MM, KR, NW, JR, MW, and GS conceived of the study. RM, DS, MM, KR, ER, NW, and JR designed and performed experiments. AD sequenced the plasmid constructs. RM wrote the manuscript. RM, AD, MW, and GS edited the manuscript.

### Conflict of interest statement

The authors declare that the research was conducted in the absence of any commercial or financial relationships that could be construed as a potential conflict of interest.
